# Molecular basis of Arginine and Lysine DNA sequence-dependent thermo-stability modulation

**DOI:** 10.1371/journal.pcbi.1009749

**Published:** 2022-01-10

**Authors:** Benjamin Martin, Pablo D. Dans, Milosz Wieczór, Nuria Villegas, Isabelle Brun-Heath, Federica Battistini, Montserrat Terrazas, Modesto Orozco

**Affiliations:** 1 Institute for Research in Biomedicine (IRB Barcelona), the Barcelona Institute of Science and Technology, Barcelona, Spain; 2 Institute of Bioengineering, School of Life Sciences, Ecole Polytechnique Fédérale de Lausanne (EPFL), Lausanne, Switzerland; 3 Department of Biological Sciences, CENUR Litoral Norte, Universidad de la República (UdelaR), Salto, Uruguay; 4 Functional Genomics Unit, Institut Pasteur de Montevideo, Montevideo, Uruguay; 5 Department of Biochemistry and Biomedicine, Faculty of Biology, University of Barcelona, Barcelona, Spain; Queen’s University, CANADA

## Abstract

We have used a variety of theoretical and experimental techniques to study the role of four basic amino acids–Arginine, Lysine, Ornithine and L-2,4-Diaminobutyric acid–on the structure, flexibility and sequence-dependent stability of DNA. We found that the presence of organic ions stabilizes the duplexes and significantly reduces the difference in stability between AT- and GC-rich duplexes with respect to the control conditions. This suggests that these amino acids, ingredients of the primordial soup during abiogenesis, could have helped to equalize the stability of AT- and GC-rich DNA oligomers, facilitating a general non-catalysed self-replication of DNA. Experiments and simulations demonstrate that organic ions have an effect that goes beyond the general electrostatic screening, involving specific interactions along the grooves of the double helix. We conclude that organic ions, largely ignored in the DNA world, should be reconsidered as crucial structural elements far from mimics of small inorganic cations.

## Author summary

Over time, scientists have proposed many different theories for the “biomolecular” origin of life. The best-known theory is the “Prebiotic or primordial soup” theory hypothesized in 1924. In this theory, nucleic acids and protein-like molecules (among others) were created from their building blocks in an aqueous environment that was supposed to be dense and enriched in organic cations and without the help of enzymatic machinery. In particular, the primordial soup was supposed to be enriched in basic, proteinogenic (Arg and Lys) and non-proteinogenic (Orn and DABA), amino acids. In such conditions, self-replication of DNA molecules was thought to occur through heat/cold cycles of duplex melting/renaturing, a process that only could have been efficient if the relative stability of AT- and GC-rich duplexes were to be similar (in contrast to what happens in normal dilute physiological conditions). Through the combination of experiments and computational simulations, we found conditions compatible with prebiotic times, where the difference in stability between AT- and GC-rich duplexes is reduced by 20 degrees in amino acids-rich solutions compared to dilute control conditions, a requirement for the self-replication of nucleic acids, the only mechanism for copying DNA information available in that early times.

## Introduction

One “dogma” originating from the early Watson-Crick models [1] is that GC-rich DNAs are more stable than the AT-rich ones [2]. Better stacking interactions combined with more favorable primary and secondary hydrogen bonds justify this difference [3–6]. However, recent experiments have challenged this “dogma” as the presence of certain organic ions was found to differentially affect the stability of A·T vs G·C base pairs [7–11] so that AT-rich DNA can be equally, or even more stable than GC-rich. These findings raise the question of the relative stability of AT- and GC-rich duplexes in the primordial soup, an environment that was supposed to be dense and enriched in organic cations [12–18], a situation that is far from the dilute aqueous solutions that are typically used to characterize biophysical properties of DNA.

In prebiotic times, DNA should have been able to replicate without the help of enzymatic machinery. We can imagine prebiotic conditions with cycles of heat/cold opening and renaturing the duplex, mimicking a protein-free “PCR-like” auto-replication process [16]. However, such a process could not have been efficient if the relative stability of AT- and GC-rich duplexes was very different, as it happens in dilute aqueous conditions. However, primordial soups was supposed to be enriched in basic amino acids [17,18], particularly the proteinogenic ones Arg and Lys, and non-proteinogenic ones such as Ornithine (synthesized at high yields in Miller-Urey experiments [19]), and L-2,4-Diaminobutyric acid (DABA). We hypothesize that such amino acids could reduce the gap between AT- and GC-rich duplexes, a requirement for the self-replication of nucleic acids, the only mechanism for copying DNA information [16]. This hypothesis links with the high prevalence of arginine and lysine interactions with DNA in known protein-DNA complexes [20] (just arginine establishes more hydrogen bonds with phosphates than all the neutral amino acids together [20]) and with the ability of small Arg and Lys peptides to condensate DNA, a requirement for defining pre-biotic phases [21], as to polymerize without the presence of enzymes large concentration of building blocks are required [22].

To test the hypothesis that basic amino acids could help to reduce GC/AT stability asymmetry, we analyse both theoretically and experimentally the role of these amino acids in modulating the structure, dynamics and stability of DNA duplexes. We demonstrate that organic ions stabilize all duplexes across a wide range of concentrations, but such stabilization is up to twice as high for the AT-rich ones. We found conditions where the difference in stability between AT- and GC-rich duplexes was reduced by 20 degrees compared to control conditions. The combination of experiments and simulations showed that the effect of organic ions cannot be explained by a simple electrostatic screening of phosphate repulsion, as usually done for small inorganic ions. In turn, it involves specific groove interactions–much more frequent in A·T than in G·C pairs–and highlights a mechanism that may not only explain prebiotic DNA replication but also contribute to our understanding of DNA replication in living cells, where the concentration of organic cations or polybasic oligopeptides can be locally high, and where interactions between cationic residues of proteins and DNA are the main responsible for the regulation of gene expression [20,23].

## Material and methods

### Preparation of the DNA duplexes and melting experiments

UV melting curves at 260 nm were measured at 2.5 μM strand concentration in buffers containing different amounts of Lysine, Arginine, Ornithine, DABA or Na^+^ and in the corresponding control buffers. The sequences investigated were purchased from Sigma Aldrich or synthesized in our lab using solid-phase DNA-synthesizer machine (only the Watson strand is reported): Seq.1 (AT-rich): 5’-TATGTATATTTTGTAATTAA-3’ and Seq. 2 (GC-rich): 5’-GTCCACGCCCGGTGCGACGG-3’. Complementary strands were heated up to 95°C and allowed to cool slowly to room temperature overnight. Melting experiments were performed in Teflon-stoppered quartz cells of 1 cm path length using a Cary 100 UV-Vis spectrophotometer at a rate of 1°C·min^-1^ from 20 to 100°C.

### Determination of thermodynamic parameters from UV denaturation studies

Melting temperatures were quantified computationally using the smoothed first derivative. Thermodynamic parameters were evaluated by measuring absorbance versus temperature curves at 1 μM, 5 μM, 20 μM and 57 μM strand concentration in Lysine, Arginine, Ornithine, Na^+^ and the corresponding control NaP 10 mM condition. Melting experiments were performed in Teflon-stoppered quartz cells of 1 cm or 1 mm path length (depending on the amount of DNA). We determined computationally the linear relationship between T_m_^-1^ and ln(Ct/2) after a least-square fitting protocol. We inferred the thermodynamic values (ΔrH_0_, ΔrS_0_ and ΔG_0,300 K_). For each case the squared correlation coefficient for the T_m_^-1^ *vs* ln(Ct/2) fit was above 0.95.

### Molecular dynamics simulations of single DNA molecules

The initial DNA duplex structures and oligonucleotides were built using Arnott-B parameters and Nucleic Acid Builder language from Ambertools 18 [24]. The duplexes were hydrated in octahedral boxes such that the box boundaries are positioned at least 1 nm away from any DNA atom. The number of ions added ensured both overall charge neutrality and a desired ratio of solute to water. For all simulated systems the ratio of solute to water was set such that the final concentration was as close as possible to 25 mM, 500 mM or 1500 mM. Note that the upper concentration range of free amino acids in solution considered here is clearly high for today’s biological conditions, but is nevertheless compatible with the total concentration of amino acids in cells (free and as part of proteins) [25], and agree with the expected large concentration of biomolecules in the primordial soup [22] where uncatalyzed polymerization of proteins and nucleic acids was possible only in highly concentrated solutions.

Systems were minimized, thermalized, pre-equilibrated and finally equilibrated for 100 ns using NPT (P = 1 atm; T = 300 K) conditions, the Nosé-Hoover thermostat [26] and the Andersen-Parrinello barostat [27]. Molecular dynamics (MD) production runs were then extended for 500 ns using state of the art simulation conditions [28,29]. Simulations were carried out using the ABC Consortium protocol (SPCE water model [30] and Smith & Dang parameters [31] for sodium and chlorine ions) [28,32] and the PARMBSC1 force-field for DNA [29,33,34]. Parameters for Lysine and Arginine were taken from a previous study by Horn *et al* [35]. For Ornithine and DABA the Lennard-Jones, bond, angle and torsional parameters were taken from Lysine, while the charges were recomputed using BCC-default procedures as implemented in the SQM program [24]. Each system was simulated using the Gromacs-5.1.4 software [36].

### Molecular dynamics simulations of large molecular systems

Large systems containing 15 AT-rich (5’- TGTATATTTTGT-3’) or 15 GC-rich (5’-CACGCCCGGTGC-3’) duplexes were simulated for 5 microseconds with each different cation. Briefly, each duplex was initially positioned at random locations in the simulation box. The duplexes were hydrated in octahedral boxes so that the box boundaries are positioned at least 1 nm away from any other DNA atom. The number of ions added ensured charge neutrality. Systems were minimized, thermalized, pre-equilibrated and finally equilibrated for 100 ns using NPT (P = 1 atm; T = 300 K) conditions. MD production runs were extended for 5,000 ns using the same simulation protocols described above for the single duplex DNA. The PARMBSC1 force-field for DNA [29,33,34] (with or without the CUFIX correction [37]), the TIP3P model to describe the water molecules [38], and Joung and Cheatham parameters for sodium and chlorine ions [39] were used. Parameters for Lysine and Arginine were taken as described above [35].

### Structural and solvent analysis

Helical parameters were computed using the Curves+/canal software [40] on full MD trajectories (with 1-ps sampling) and standard helical nomenclature (see S1 Fig) [41]. The structural fluctuations of the helical parameters were assessed using the data series and the Kullback–Leibler divergence. Lys/Arg and other ion densities were obtained using curvilinear helicoidal coordinates for each snapshot of the simulations with respect to the instantaneous helical axis, as implemented in Curves+/canion software [42–44]. The ions densities were computed in both minor and major grooves using the angles and base pair definitions previously published [29]. Convergence analysis was done to ensure that the densities were derived from MD at equilibrium (S2 Fig). The reference atoms to follow Lys or Arg in the curvilinear helicoidal space were Cα/NZ and Cα/CZ, respectively. For Essential Dynamics [45], the eigenvalues/eigenvectors were computed using the Gromacs *covar* and *anaeig* functions with default options. The first 10 eigenvalues were computed using the same reference structure for all AT- or GC-rich DNA duplexes in the different ionic conditions. DNA stiffness computation was performed as described elsewhere [46–48].

### Free energy calculations

The reversible work associated with the change A↔G and T↔C in single stranded and duplex DNA were computed using as model of the single-stranded DNA tetranucleotides, d(CXXT), where the two central bases were alchemically mutated: ApA to GpG, ApT to GpC, TpA to CpG, TpT to CpC respectively. The double-stranded systems contained a dsDNA decamer with a sequence 5’-CATCXXTGCA-3’, where the four central alchemical XpX base pairs were mutated in both strands in an identical fashion. Single-stranded and duplex systems were surrounded by water adding NaCl, ArgCl or LysCl to guarantee the desired concentration (see above). Calculations performed with standard PARMBSC1 were repeated using CUFIX corrections, finding only moderate deviations (see Results). Reversible works for single stranded (ssDNA) and duplexes (dsDNA) were combined using standard thermodynamic cycles (see S3 Fig) to determine the impact of the ionic atmosphere on the relative stability of the AT and GC- duplexes.

To sample the equilibrium ensembles in physical endpoints, 12×50 ns (dsDNA) or 4×50 ns (ssDNA) equilibrium runs per system were performed, the replicas initialized from different random positions of the co-solute to enhance configurational sampling. Then, an ensemble of non-equilibrium slow-growth trajectories was generated using a custom Python script (gitlab.com/KomBioMol/crooks), with 184 250-ps runs produced from each endpoint. For simulations using the CUFIX correction, each Crook’s non-equilibrium simulation was preceded by additional 50 ps re-equilibration to allow for relaxation due to minor changes in force field parameters. Finally, kernel density estimation (as implemented in Python’s scikit-learn) [48] was used to calculate the intersection of work probability densities that coincides with the free energy estimate, according to the Crooks theorem [49].

## Results and discussion

### Melting experiments reveal a strong and specific stabilizing effect of cationic amino acids on DNA

**Fig 1:**
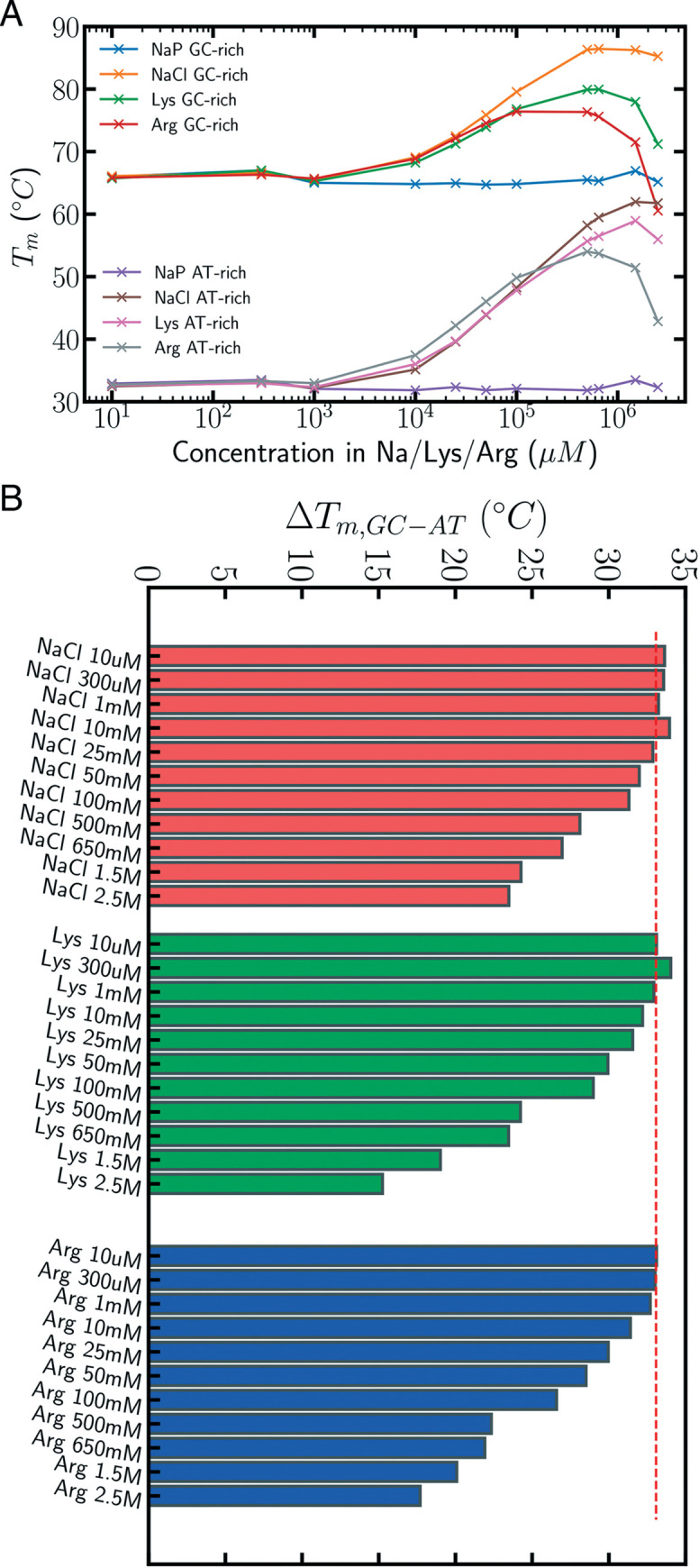
Zwitterionic amino acids Lysine and Arginine stabilize differentially AT- vs GC-rich DNA duplexes. A) Tm for the different cationic conditions and concentrations (average between two replicas–std < 1% of the average for all samples). For the control NaP conditions, the concentration is constant at 10 mM. B) ΔTm represents the difference in melting temperature between CG-rich and AT-rich duplexes. The red dashed line represents the mean difference in melting temperature across NaP controls between GC-rich and AT-rich duplexes (std < 2% mean for all conditions).

Melting experiments (Fig 1A) demonstrate that proteinogenic amino acids (Arg and Lys) stabilize DNA with respect to the default conditions (10 mM Na protonated, Na-P) at cation concentrations above 1 mM. At very high concentrations of cations (above 650 mM) saturation is achieved (Fig 1A). The cationic stabilizing effect is more intense, at each cation concentration, for the AT-rich compared to the GC-rich DNA duplex (Fig 1). Thus in control conditions, the GC-rich duplex shows a melting temperature about 35 degrees higher than the AT-rich duplex, but this difference is reduced by c.a. 15 degrees in the presence of high concentrations of Lys or Arg. Note that such a dramatic modulation in the relative stability of duplexes AT or GC-rich is not found when NaP concentration is increased (Fig 1B), indicating that the absolute and relative stabilization cannot be fully explained by a simple ion screening effect. Thermodynamic analysis of the data reveals that while the cation-induced stabilization of the duplexes has an enthalpic origin, the differential AT/GC stabilization has an entropic origin probably related to water release upon cation binding (see below and S4 Fig).

**Fig 2:**
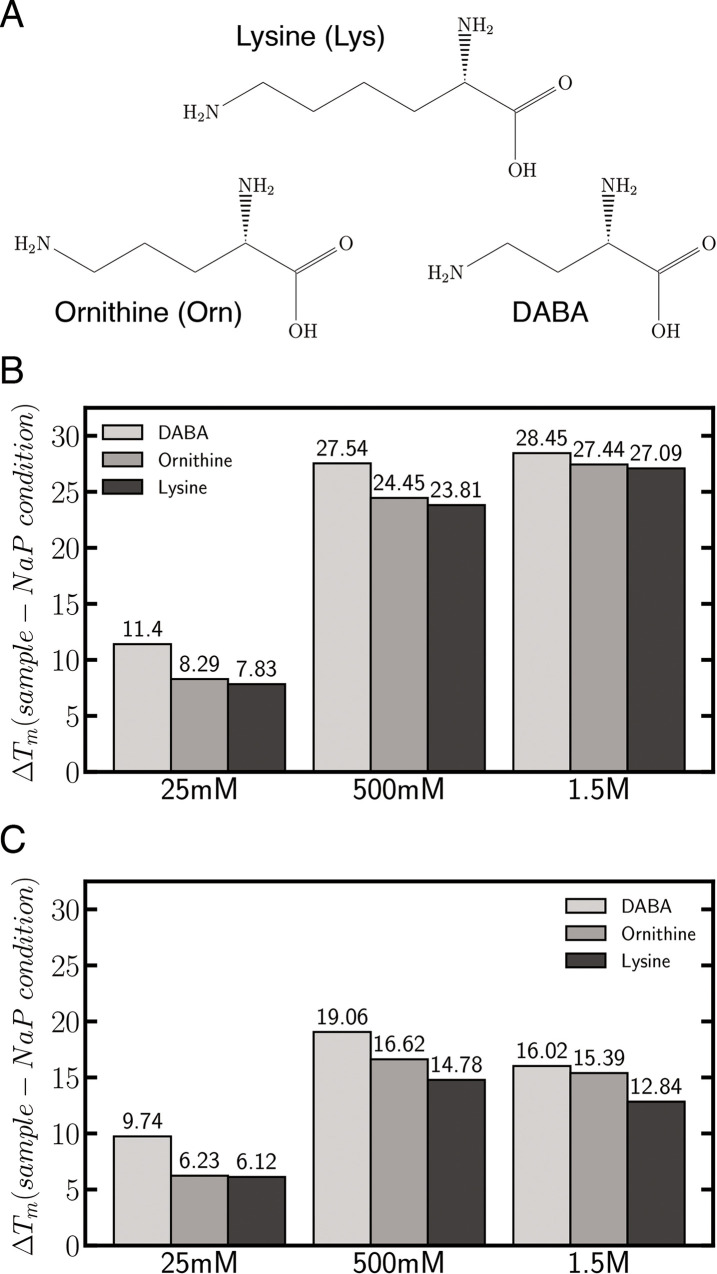
Lysine and derivatives stabilize AT- vs GC-rich DNA duplexes differentially depending in the length of their carbonated side chain. A) Scheme of Lysine and Lys-derivative structures. B) Melting temperatures relative to NaP buffer (ΔTm) of AT-rich duplexes in the presence of Lysine and Lys-derivatives. C) Melting temperatures relative to NaP buffer (ΔTm) for GC-rich duplexes in the presence of Lysine and Lys-derivatives (average between two replicas—std < 3% of the average for all samples).

We then explored whether the results obtained for these two duplexes could be extended to others with a variable number of AT/GC pairs, as well as to other amino acids present in the primordial soup. Results in S2 Table demonstrate that, indeed, our conclusions can be extrapolated to any DNA duplex and that basic amino acids reduce dramatically the stability difference between AT- and GC-rich DNAs. For example, in the presence of ArgCl 650 mM, an AT-rich duplex (10% GC) displays the same stability as a GC-rich duplex (60% GC). Furthermore, we repeated the melting experiments using Ornithine (Orn) and L-2,4-Diaminobutyric acid (DABA) (Fig 2A), and obtained profiles similar to that of Lys (stabilizing AT-rich duplexes more than GC-rich ones), with the stabilizing effect getting stronger as the hydrocarbon chain shortens (Lys<Orn<DABA; see Fig 2B and 2C) with a maximum of stability for Na^+^. This trend is likely to reflect the increase in positive charges in the grooves as a consequence of the reduction of the excluded volume generated by the aliphatic chain. In summary, experimental results strongly suggest that a reasonably high concentration of amino acids in the primordial soup could lead to conditions where the critical temperature of folding and unfolding is less dependent from the sequence, favouring the self-replication of DNA. However, even at high concentrations, Arg and Lys are not able to make equally stable GC- and AT- rich duplexes; other small bioorganic cations [7] and small polycationic peptides [21,50] can probably complete the stability equalization of GC- *vs* AT- rich sequences.

### The origin of the amino acid stabilization of DNA

**Fig 3:**
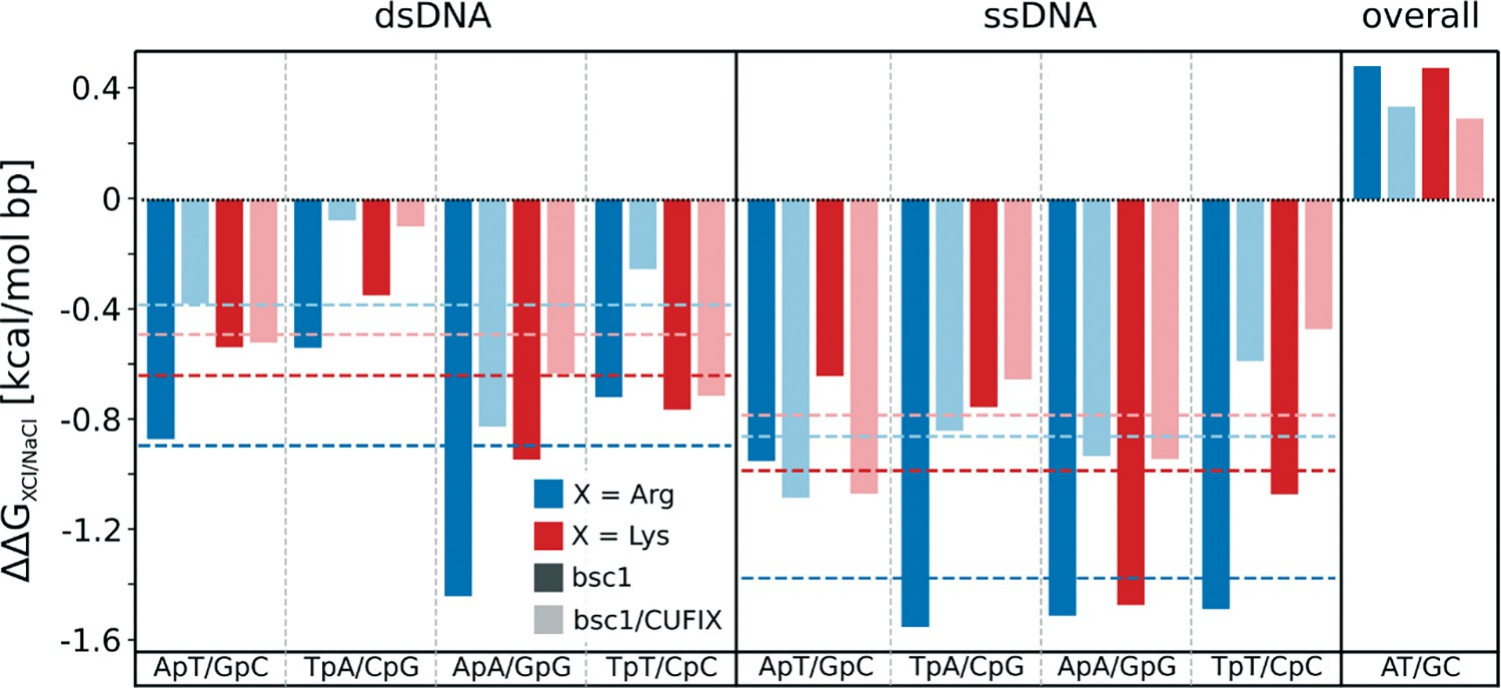
Changes in the relative stability of AT/GC DNAs induced by the presence of 500mM of ArgCl of LysCl (all values referred to the 500 mM NaCl). The values for ssDNA were obtained by mutating pairs of nucleotides: d(ApA)→d(GpG), d(ApT)→d(GpC), d(TpA)→d(CpG) and TpT→CpC in a d(CXXT) tetramer. The values for the duplex (dsDNA) were obtained by mutating A·T→G·C in a decamer (dCATCXXTGCA), with double base-pair mutations at the XX positions performed in the same fashion (values are reported per single A·T/G·C substitution). A positive value of ΔΔG_XCl/NaCl_ (ssDNA) means that the change from NaCl to organic salt favours the stability of A/T containing single strands (with respect to those containing G/C). A positive value of ΔΔG_XCl/NaCl_ (dsDNA) means that the change from NaCl to organic salt favours the stability of A/T containing duplexes (with respect to G/C containing ones). The “overall” values (last column) correspond to the effect of organic salt (taken as reference value 500 mM NaCl) on the difference in the folding free energy (ss→sd) of AT and GC DNAs; values being referred to a single A·T/G·C substitution. A positive value of ΔΔG_XCl/NaCl_ (overall) means that the change from NaCl to organic salt favours the folding of AT-rich duplexes (with reference to GC-rich ones). A one-sample t-test was performed to test significance. All calculations were performed with and without CUFIX corrections, dark and light colours respectively. See Material and Methods and S3 Fig for details on the thermodynamic cycles used.

To gain understanding of the reasons of the sequence-dependent stabilizing effect of amino acids on DNA, we performed free energy calculations to estimate the change in stability of AT- vs GC-rich duplexes as a function of the cationic environment (see Material and Methods). The results, shown in Fig 3, demonstrate that GC-rich single stranded DNAs (ss) increase their stability with respect to AT-rich ones when NaCl is substituted by LysCl or ArgCl (500 mM ion concentration), suggesting that organic cations solvate GC-rich single stranded DNAs better than AT-rich ones (as compared to Na^+^). The same calculations for double stranded (ds) DNA provide different results depending on the sequence context, suggesting that local structural effects have a major role in modulating cation-water-DNA interactions (see Fig 3). However, by averaging over different sequence environments and subtracting the relative free energy estimates for ds and ss DNAs, we can conclude (see Fig 3; right panel) that the substitution of NaCl by salts of basic amino acids (specially ArgCl) leads to an overall significant stabilization of the folded state in AT- vs GC-rich DNAs (*i*.*e*. an increase in the relative free energy of unfolding). Contrary to our original expectations based on comprehensive studies of phosphate-amino interactions by other groups [23,37] the impact of introducing the CUFIX correction (see Material and Methods) is small, reinforcing our confidence in the simulations.

Our free energy calculations (Fig 3) agree with experimental results, showing that basic amino acids indeed reduce the gap in stability between AT- and GC-rich duplexes. Part of this stabilizing effect can be explained considering that basic amino acids stabilize GC-rich single stranded DNA (the unfolded state) more than in case of AT-rich DNA, an effect that is similar for Arg and Lys as both can effectively compete with Watson-Crick hydrogen bonding. However, the same calculations also suggest a differential effect of the amino acid on the intrinsic duplex stability and a dependence on the sequence context. This point towards the presence of specific interactions of Arg^+^ and Lys^+^ with the DNA duplex, a point that was analysed in the equilibrium MD simulations of AT- and GC-rich duplexes (see Material and Methods).

**Fig 4:**
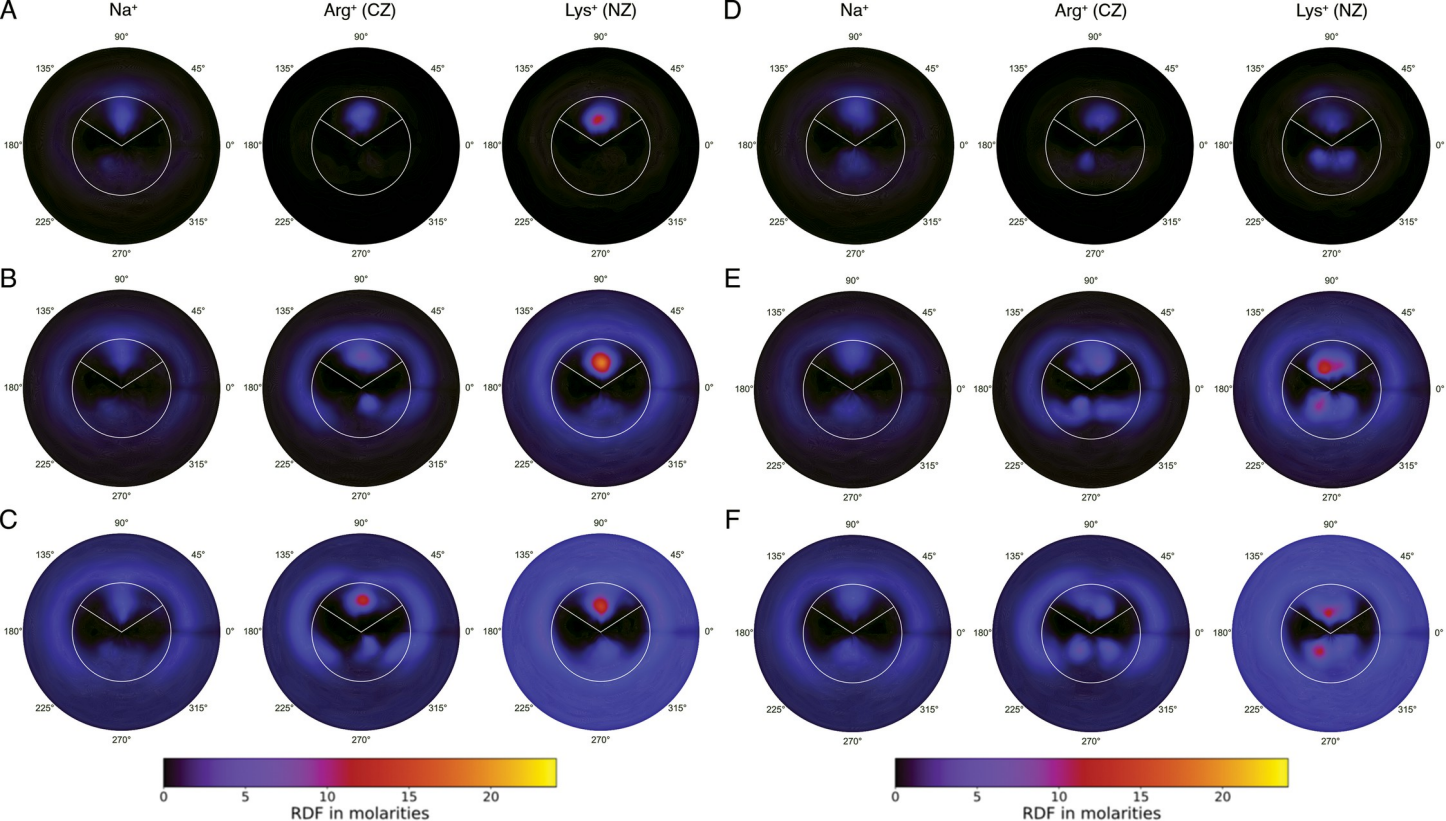
Distributions of the cations around AT-rich and GC-rich DNA duplexes. A) Averaged radial-angular dependence of Na^+^, Arg^+^ (CZ atom) and Lys^+^ (NZ atom) from AT-rich duplexes at 25 mM concentration. The centre of each circle represent the average axis of the DNA-duplex. The minor-groove extends from 33° to 147°, while the major-groove extends from 147° to 33° (in the trigonometric direction). The white circle delimits the inner space of both minor and major grooves (localized 10.25 Å from the DNA average axis). B) Same as (A) for simulations done at 500 mM. C) Same than (B) at 1.5 M concentration. D), E), F), same as (A), (B), and (C) for GC-rich DNA duplexes respectively. Note that in all cases the total radial cut-off is 20.5 Å.

Integration of the density of cations (Na^+^, Arg^+^ or Lys^+^) along helical coordinates shows a large concentration (more than 120 times the concentration in the bulk solvent) of basic amino acids in the grooves compared to Na^+^ at equivalent ionic strength (Fig 4). Interestingly, while the increase in NaCl concentration from 25 mM to 500 mM introduces only changes in the concentration of Na^+^ outside the grooves (suggesting ion saturation), the same increase in Arg^+^ or Lys^+^ concentration leads to a clear intensification in the concentration of the amino acids in the minor groove. As expected from experimental results above, further increase in Arg+ or Lys+ concentration to 1.5 M does not lead to significant changes in the number of amino acids in the grooves as they reach saturation.

The amino acids fitting the grooves tend to form strings (see amino acids along the grooves in S5 and S6 Figs), displacing water molecules from the first solvation shell. This is clear when analysing water density around DNA duplex at different concentrations of NaCl, ArgCl and LysCl (S7 Fig). As expected, bulkier amino acids liberate more water molecules from the first solvation shell, something that is not so evident to the smaller Na+ which can coexist better with water molecules, while water molecules are mainly displaced from the minor groove in the AT-rich sequence, waters around the backbone and the grooves are significantly depleted at high amino acid concentrations for the GC-rich duplex. Clearly, the removal of ordered waters around DNA increases water entropy contributing to the stability of the duplexes at high concentration of basic amino acids.

As shown in Fig 4, the highest concentration of amino acids appears in the minor groove, especially in AT-rich duplexes, due probably to the narrow minor groove and the richness of hydrogen bond acceptors at the bottom of AT regions of a DNA duplex. This pattern of ion density is similar to that found for equivalent ionic strengths in the control NaCl simulations, but with more marked peaks (S8 Fig). Interestingly, as salt molarity increases, the amino acids also appear along the major groove, in particular for the GC-rich duplexes. As noted above, when increasing ion concentration water densities follow the opposite trend than the ions (see S7 Fig), confirming the tight coupling between ion binding and water release.

### Structural impact of basic amino acids in AT- and GC-rich DNA-duplexes

**Fig 5:**
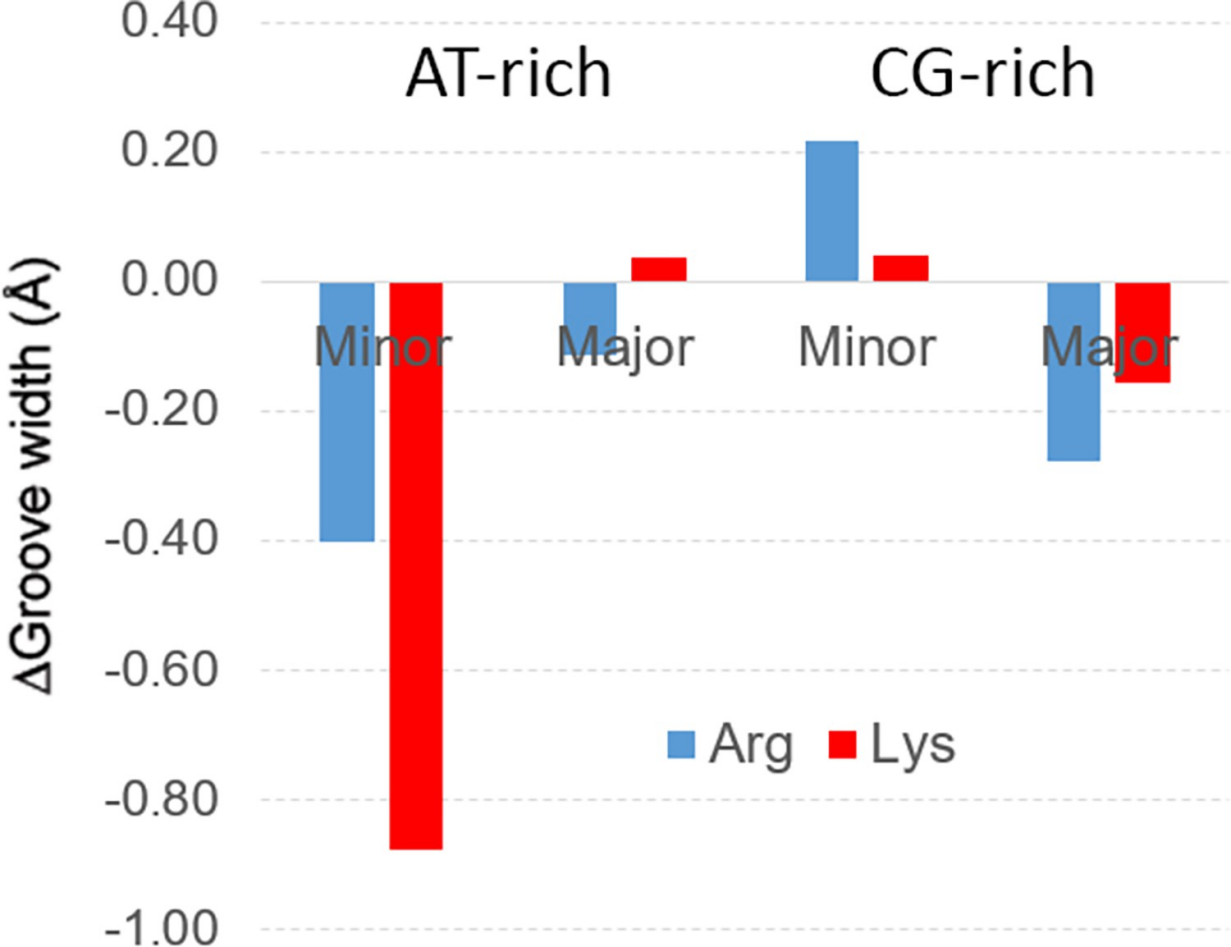
Variation in major and minor groove width. Average grooves width of AT-rich and GC-rich duplexes when the 500 mM (NaCl) buffer is replaced by 500 mM LysCl (Lys, red) or ArgCl (Arg, blue) buffers (negative number means groove width narrowing when moving from NaCl to LysCl or ArgCl).

The substitution of Na^+^ by Lys^+^ or Arg^+^ has a non-negligible effect on DNA geometry. Kullback-Leibler divergence (D_KL_) calculations (see Material and Methods and S9 Fig) show that the parameters more sensitive to the substitution of Na^+^ by organic cations are groove dimensions, base pair helical parameters coupled with groove geometry (such as slide and shift) and parameters informing on the distortion of hydrogen bond and stacking related to a loss in base pair planarity (shear, stagger, and twist). Note that although shear and stagger are intra base pair parameters, they both represent a translation of the base plane in the x- or z-axis respectively (see S1 Fig), and hence, their relative movements respect to nearest neighbours base pairs, affect π-π interactions and consequently the stacking energy. Changes in base pair parameters are explained by the reduced ability of organic cations to compete for intramolecular hydrogen bond compared to small Na^+^ ions (see S10 Fig). However, the most prevalent structural changes coupled with the substitution of NaCl by ArgCl or LysCl are related to the grooves. This effect, which is typical of groove binders, is triggered by the screening of phosphate repulsion and the formation of van der Waals contacts between the walls of the groove and the amino acids. The minor groove is the most affected one by the presence of charged amino acids in AT-rich duplexes displaying a clear narrowing effect, while in GC-rich duplexes the largest changes are detected in the major groove (Fig 5 and S3 Table), where the concentration of organic cations leads to a small but consistent widening when going from 0.5 M to 1.5 M (S3 Table). Overall Arg and Lys structural changes can be easily explained by looking at ion densities in AT- and GC- duplexes in Fig 4 and by considering the well-known tendency of the grooves to coordinate cations [51,52], which displace water and screen phosphate repulsion.

### The impact of ionic atmosphere on the local and global dynamics of DNA

**Fig 6:**
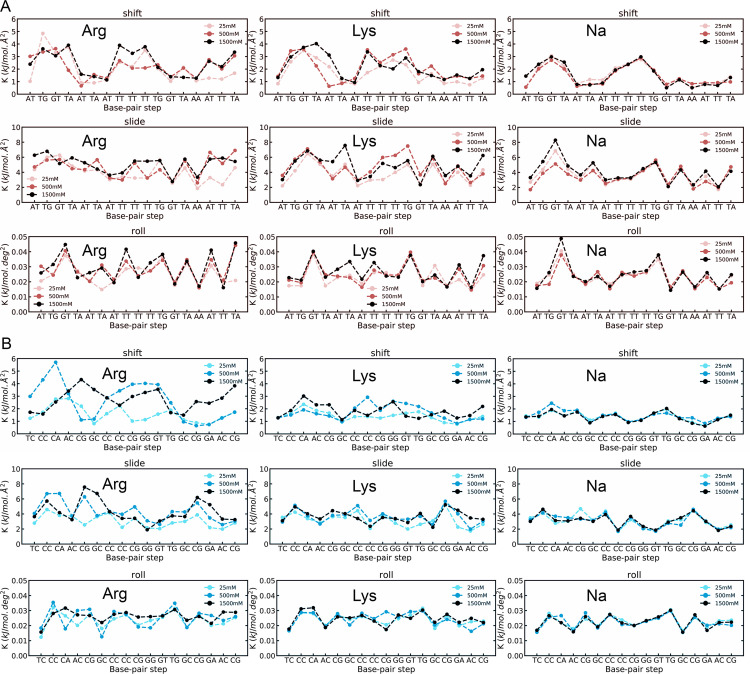
Selected translational and rotational force constants associated to each basepair steps. A) Pure (diagonal values) of Shift-Shift, Slide-Slide and Roll-Roll sequence-dependent force constants for AT-rich sequences (red-ish series). B) Same as (A) for GC-rich sequences (blue-ish series).

The increase of ionic strength leads to a general increase in stiffness, as particularly visible in local helical parameters mainly responsible for DNA bending (Fig 6) that were also previously identified as the most polymorphic degrees of freedom (e.g. twist) [28,42,32]. This effect is more evident when the cations in solution are amino acids, which reduce the conformational freedom of DNA. Interestingly, while the sequence-dependent stiffness profile does not change much when NaCl concentration increases from 25 mM to 1.5 M, significant changes in the stiffness profiles were detected along the same concentration range for ArgCl and LysCl (see Figs 6 and S11). Remarkably, not only local variations of stiffness are higher, but they also extend for large segments. We computed an overall stiffness value for each bp and a normalized overall stiffness value for the complete DNA molecule by averaging the base pairs values (S4 Table) [53]. In doing so, we found that duplexes got stiffer with the increasing of the concentration of organic cations. These findings agree with an effect of basic amino acids that cannot be explained solely by a general increase in ionic strength.

**Fig 7:**
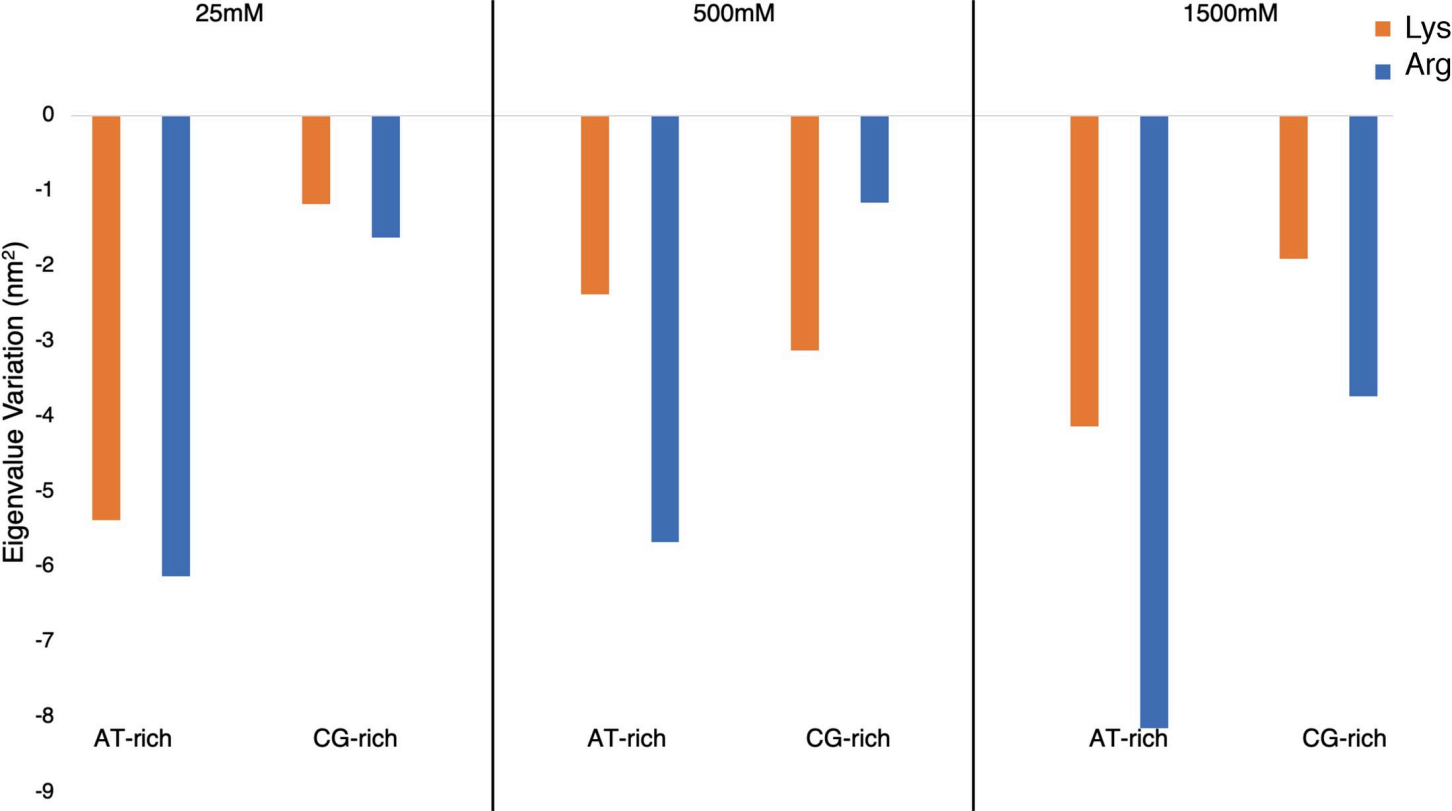
Variation in Eigenvalues. Difference between the sum of the first eigenvalues for AT-rich and CG-rich duplexes with Arg and Lys respectively, and the sum of the same duplex, at the same Na concentration.

The global dynamics of the duplexes was also compared by analysing their essential dynamics. In Fig 7, we report the sum of the first eigenvalues relative to equivalent NaCl conditions. In general, organic cations affect more the AT-rich than the GC-rich DNA duplexes. Going from Na to Arg the AT-rich duplexes increase in stiffness (see Fig 7), with a higher effect at high concentration of Arg (1500 mM). GC-rich duplexes show a similar tendency, but with smaller cation-induced global flexibility changes. Lys seems to have a lower effect on the duplex flexibility than Arg as its concentration increases. Contrary to the sizeable change in the extend of nucleic acid flexibility induced by organic cations, no significant changes in the nature of the movements have been detected as shown in the normalized overlap of the conformational ensembles (see S5 Table).

### Crowding and concentration effects

**Fig 8:**
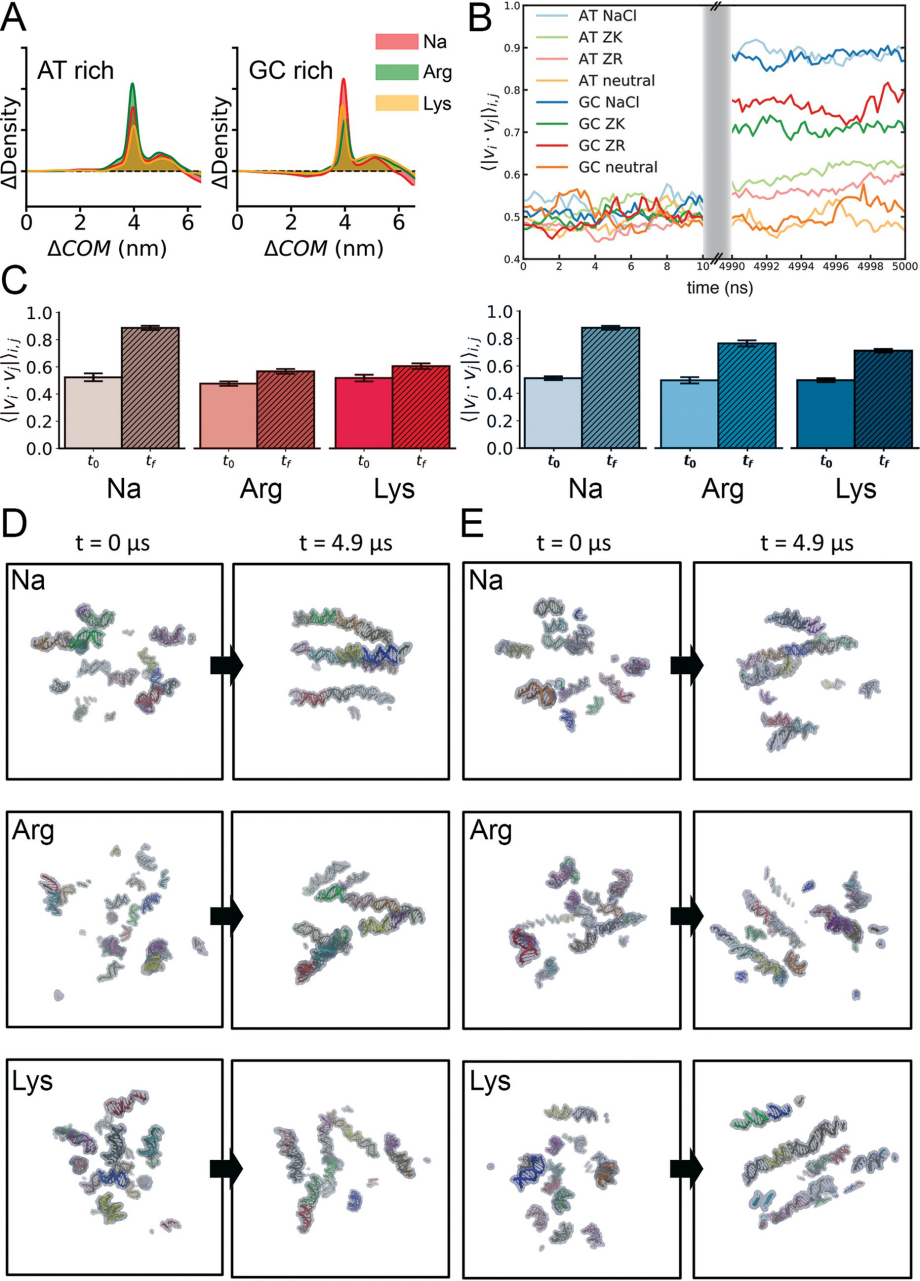
Structure and dynamics of large molecular systems simulated. A) Difference between the relative Center-of-Mass (ΔCOM) displacement of the 15 DNA duplexes in each of the NaCl, ArgCl and LysCl system respect to the electroneutrality case. B) Pair-wise cross-correlation coefficient between vectors that represents the helical axe of each DNA duplex along the total simulated time. First and last 10 ns are shown. C) Pair-wise cross-correlation coefficient between helical axes of each DNA duplex for AT-rich (red-ish series) *vs* GC-rich (blue-ish series). D) Initial and one representative “final” structure obtained from the corresponding MD trajectories of AT-rich systems. Each duplex is depicted in cartoon representation using different colours. E) Same as (D) for GC-rich system simulated.

To explore the effect of cations on DNA-DNA interactions, we simulated large molecular systems containing 15 copies of AT- or GC-rich duplexes with each ions respectively (see Material and Methods) at the multi-microsecond timescale. Large concentration of cations (500mM of either amino acids or Na^+^) leads to the formation of a microenvironment of high DNA density as noted in the shortening of the DNA-DNA distance (Fig 8A and 8B), and interestingly to the spontaneous formation of pseudo-fibers with a clear alignment of the DNA main axis and strong correlation in their movements (Figs 8B–8E and S12). Local concentration of cations in these dense DNA environments can be very high, maximizing ion-induced effects (S12 Fig). Condensation/fiber generation effects are in general more evident in GC-rich DNAs than in AT-rich ones, in agreement with differential ion interaction properties described above. However, we do not detect here any systematic difference between the effect of organic and inorganic ions, suggesting that we are finding a general cation-induced condensation of DNA that can be universal in all conditions were DNA compaction is required. However, higher DNA concentration phases could require the presence of organic polycations [21,50].

## Conclusions

The interaction of inorganic cations with DNA has been largely studied using both high resolution structural techniques and accurate MD simulations, which have described the preferential sites of cation-binding and even the dynamics of ion interchange [29,42–44,52,54–56]. However, little is known about the impact of organic cations, particularly, of basic amino acids that were present in the primordial soup. Previous analyses using organic cations [50,57,58] suggested to us that basic amino acids, positively charged at neutral pH, might play a sequence-specific stabilizing effect, different from that of inorganic ions, and thus alter the preferential stability of AT- and GC- rich duplexes, a requirement for efficient auto-replication of DNA duplexes. Results reported here support this hypothesis: moving to an environment rich in basic amino acids not only increases the stability of DNA duplexes, but also decreases quite significantly, the stability gap between AT- and GC- rich duplexes, something that does not happen in the presence of equivalent concentration of inorganic cations. The physical reasons of the sequence-dependent stabilization of basic amino acids for AT- vs GC- rich duplexes (with respect to Na) are double: on one hand Lys and Arg fits very well into the AT-rich minor groove of DNA and on the other hand, both amino acids stabilize preferentially the unfolded state of the GC-rich DNA, mostly due to the interactions with guanine in the N7-side [20]. The effect of basic amino acids is evident at a concentration range compatible with primordial soup conditions, but it is insufficient to explain a full equalization of the stability of GC- and AT- duplexes. Clearly, other small ligand, most likely small cationic peptides could contribute to make equally possible replication of AT- and GC- rich DNA duplexes.

Present results provide clues on the mechanisms of early DNA replication in prebiotic conditions, where DNA was supposed to be embedded in a dense environment of amino acids and small polycationic peptides, which could not only reduce the GC- vs AT- duplex stability gap, but also produce significant condensation which could increase the local concentration to facilitate polymerization reactions [21,50]. The groove-preference of the basic amino acids strongly suggest that the Arg+ / Lys+ equalizing effect is DNA specific and should not affect, at least to the same extend the RNA duplex, where the equivalence in AT- and GC- duplex stability should be achieved by other biomolecules, like sugars. Finally, it is worth noting that the study presented here even designed to gain insight into non-enzymatic DNA replication in prebiotic conditions, may also help to understand better the interplay between DNA stability, structure and compaction in virus capsids, bacterial nucleoids and cell nuclei, where histone tails and/or a myriad of effector proteins and peptides generate an atmosphere rich in polybasic, Arg- and Lys-rich tails [20,21,23].

## Supporting information

S1 Table Details of each simulation performed in this work with relative ID in the BigNASim database. (PDF)

S2 Table Melting temperatures in NaP conditions, 650 mM of NaCl and 650 mM of ArgCl. (PDF)

S3 Table Sequence-averaged major and minor groove width values for AT-rich and CG-rich sequences. (PDF)

S4 Table Averaged overall stiffness values for the entire duplex (in kJ/(mol). (PDF)

S5 Table Normalized overlap of the covariance matrices computed using the 10 first eigenvectors. (PDF)

S1 Fig Helical parameters approved by the Cambridge convention [1]. Adapted from Lu and Olson 2003 [2].
(TIF)

S2 Fig Convergence of the cations populations around DNA. Left figure. Inner minor-groove Right figure. Inner major-groove. Note that here 2,500 frames are equivalent to 5 microseconds.
(TIF)

S3 Fig Thermodynamic cycles used to compute the change in folding free energy related to the the AT/GC content. The estimates obtained in NaCl, ArgCl, or LysCl can be manipulated to obtain the differential impact of salt in AT vs GC duplexes.
(TIF)

S4 Fig The zwitterionic Lysine and Arginine alters the thermo- dynamic parameters of the DNA duplex melting reaction in a GC- content—dependent manner. TOP: ΔΔrGcondition−NaP,300°K for each cationic condition. MIDDLE: ΔΔrHcondition−NaP for each cationic condition. BOTTOM: ΔΔrScondition−NaP for each cationic condition. All the values are related to the direct DNA duplex melting reaction.
(TIF)

S5 Fig Detail of the arginine localization around AT- (TOP) and GC-rich DNA (BOTTOM). 25mM MD simulations were used to extract this Figure.
(TIF)

S6 Fig Lysine localization around AT- (TOP) and GC-rich DNA (BOTTOM). 25mM MD simulations were used to extract this Figure.
(TIF)

S7 Fig Distributions of water molecules around AT-rich and GC-rich DNA duplexes. A) Averaged radial-angular dependence of OW atom from AT-rich duplexes at 25 mM concentration. The centre of each circle represent the average axis of the DNA-duplex. The minor-groove extends from 33° to 147°, while the major-groove extends from 147° to 33° (in the trigonometric direction). The white circle delimits the inner space of both minor and major grooves (localized 10.25 Å from the DNA average axis). B) Same as (A) for simulations done at 500 mM. C) Same than (B) at 1.5 M concentration. D), E), F), same as (A), (B), and (C) for GC-rich DNA duplexes respectively.
(TIF)

S8 Fig Radial distribution function of the cations around AT- (A) and GC-rich (B) DNA duplexes considering the Na, CZ or NZ atoms. The x-axis represents the distance from DNA average axis in Å ranging from 0 to 10.25. The sequence in place of the DNA average axis is the arbitrary Watson sequence as written in Material and Methods. MajG: major-groove. MinG: minor-groove.
(TIF)

S9 Fig Zwitterionic Lysine and Arginine affects the structural parameters of the DNA in a GC-content and cation-type—dependent manner. KL-divergence between helical parameters for each cationic conditions at 1500mM ion concentration for AT- (A) and GC-rich (B) DNA duplexes. Note that only the helical parameters that showed some changes are reported.
(TIF)

S10 Fig Distribution plots of the number of intra-molecular DNA hydrogen bonds in all the cationic and GC-content conditions tested. Only the final 200ns of the MD trajectories were considered.
(TIF)

S11 Fig Selected translational and rotational force constants associated to each basepair steps. A) Pure (diagonal values) of Rise-Rise, Tilt-Tilt and Twist-Twist sequence-dependent force constants for AT-rich sequences (red-ish series). B) Same as (A) for GC-rich sequences (blue-ish series).
(TIF)

S12 Fig Structure and dynamics of large molecular systems simulated. A) Mean-Square-Displacement (MSD) along the simulated time. Note that the Diffusion Coefficient was computed from 3.25 μs to 3.5 μs (linear regime between the vertical dashed lines). B) Pair-wise cross-correlation coefficient between vectors that represents the helical axe of each DNA duplex along the total simulated time. First and last 10 ns are shown. C) Initial and one representative “final” structure obtained from the corresponding MD trajectories of AT-rich systems under electroneutrality conditions. Each duplex is depicted in cartoon representation using different colors. D) Same as (C) for GC-rich systems.
(TIF)
