# Supplementary material for: Molecular basis of Arginine and Lysine DNA sequence-dependent thermo-stability modulation
Source: PLoS Comput Biol. 2022 Jan 10;18(1):e1009749. doi: 10.1371/journal.pcbi.1009749 (PMC8782489; doi:10.1371/journal.pcbi.1009749)
Supplement: S1 Table — (PDF) [file pcbi.1009749.s001.pdf]

S1 Table. Details of each simulation performed in this work with relative ID in the BigNASim database.

Single duplex						
Duplex	Cation	Concentration	Time	#H2O	#Cations	ID
AT-rich	Na	25 mM	400 ns	19352	47	AT-rich_Na_25mM
AT-rich	Lys	25 mM	400 ns	19299	9	AT-rich_Lys_25mM
AT-rich	Arg	25 mM	400 ns	19286	9	AT-rich_Arg_25mM
AT-rich	Orn	25 mM	400 ns		9	AT-rich_Orn_25mM
AT-rich	DABA	25 mM	400 ns		9	AT-rich_DABA_25mM
GC-rich	Na	25 mM	400 ns	19359	47	GC-rich_Na_25mM
GC-rich	Lys	25 mM	400 ns	19313	9	GC-rich_Lys_25mM
GC-rich	Arg	25 mM	400 ns	19295	9	GC-rich_Arg_25mM
GC-rich	Orn	25 mM	400 ns		9	GC-rich_Orn_25mM
GC-rich	DABA	25 mM	400 ns		9	GC-rich_DABA_25mM
AT-rich	Na	500 mM	400 ns	19016	215	AT-rich_Na_500mM
AT-rich	Lys	500 mM	400 ns	17765	177	AT-rich_Lys_500mM
AT-rich	Arg	500 mM	400 ns	17582	177	AT-rich_Arg_500mM
AT-rich	Orn	500 mM	400 ns		177	AT-rich_Orn_500mM
AT-rich	DABA	500 mM	400 ns		177	AT-rich_DABA_500mM
GC-rich	Na	500 mM	400 ns	19023	215	GC-rich_Na_500mM
GC-rich	Lys	500 mM	400 ns	17755	177	GC-rich_Lys_500mM
GC-rich	Arg	500 mM	400 ns	17541	177	GC-rich_Arg_500mM
GC-rich	Orn	500 mM	400 ns		177	GC-rich_Orn_50mM
GC-rich	DABA	500 mM	400 ns		177	GC-rich_DABA_500mM
AT-rich	Na	1500 mM	400 ns	18310	568	AT-rich_Na_1500mM
AT-rich	Lys	1500 mM	400 ns	14431	530	AT-rich_Lys_1500mM
AT-rich	Arg	1500 mM	400 ns	13777	530	AT-rich_Arg_1500mM
AT-rich	Orn	1500 mM	400 ns		530	AT-rich_Orn_1500mM
AT-rich	DABA	1500 mM	400 ns		530	AT-rich_DABA_1500mM
GC-rich	Na	1500 mM	400 ns	18317	568	GC-rich_Na_1500mM
GC-rich	Lys	1500 mM	400 ns	14421	530	GC-rich_Lys_1500mM
GC-rich	Arg	1500 mM	400 ns	13819	530	GC-rich_Arg_1500mM
GC-rich	Orn	1500 mM	400 ns		530	GC-rich_Orn_1500mM
GC-rich	DABA	1500 mM	400 ns		530	GC-rich_DABA_1500mM
15 duplexes - condensation assay MD (with CUFIX)						
AT-rich	Neutral	500 mM	5 µs	125229	330 (Na)	AT-rich_crowd_neut_500mM
AT-rich	Na	500 mM	5 µs	123605	1142	AT-rich_crowd_Na_500mM
AT-rich	Lys	500 mM	5 µs	114861	1142	AT-rich_crowd_Lys_500mM
AT-rich	Arg	500 mM	5 µs	113646	1142	AT-rich_crowd_Arg_500mM
GC-rich	Neutral	500 mM	5 µs	125202	330 (Na)	GC-rich_crowd_neut_500mM
GC-rich	Na	500 mM	5 µs	123578	1142	GC-rich_crowd_neut_Na_500mM
GC-rich	Lys	500 mM	5 µs	115011	1142	GC-rich_crowd_Lys_500mM
GC-rich	Arg	500 mM	5 µs	113692	1142	GC-rich_crowd_Arg_500mM
