# Supplementary material for: Molecular basis of Arginine and Lysine DNA sequence-dependent thermo-stability modulation
Source: PLoS Comput Biol. 2022 Jan 10;18(1):e1009749. doi: 10.1371/journal.pcbi.1009749 (PMC8782489; doi:10.1371/journal.pcbi.1009749)
Supplement: S2 Table — (PDF) [file pcbi.1009749.s002.pdf]

S2 Table. Melting temperatures in NaP conditions, 650 mM of NaCl and 650 mM of ArgCl.

		NaP	NaCl		Arg	
%GC	Sequence	T_m (°C)	T_m (°C)	ΔT_m (°C)	T_m (°C)	ΔT_m (°C)
10	d(TATGTATATTTTGTAATTAA)	34	60	26	54	20
20	d(TATTTCATTTGTTCTGTAAT)	37	64	27	57	20
30	d(CATTTCCTTTGTTCTGTAAT)	40	67	27	60	20
40	d(CGTTTCCTTTGTTCTGTAGT)	44	70	26	63	19
50	d(CGTTCCCTTTGTTCTGGAGT)	48	72	24	65	17
60	d(GTCCACTCTCGGTGCAACTG)	55	79	24	69	14
70	d(GTCCACGCTCGGTGCGACTG)	63	84	21	73	10
80	d(GTCCACGCCCGGTGCGACGG)	67	87	20	75	8
