# Supplementary material for: Molecular basis of Arginine and Lysine DNA sequence-dependent thermo-stability modulation
Source: PLoS Comput Biol. 2022 Jan 10;18(1):e1009749. doi: 10.1371/journal.pcbi.1009749 (PMC8782489; doi:10.1371/journal.pcbi.1009749)
Supplement: S3 Table — (PDF) [file pcbi.1009749.s003.pdf]

S3 Table. Sequence-averaged major and minor groove width values for AT-rich and CG-rich sequences.

	Arg	sd	Lys	sd	Na	sd
			Major groove width			
AT-rich 0.5M	12.37	1.45	12.52	1.35	12.49	1.51
AT-rich 1.5M	12.52	1.22	12.31	1.32	12.28	1.44
GC-rich 0.5M	11.56	1.48	11.68	1.38	11.84	1.57
GC-rich 1.5M	12.12	1.43	11.94	1.50	11.75	1.48
			Minor groove width			
AT-rich 0.5M	4.93	1.05	4.45	1.15	5.33	1.41
AT-rich 1.5M	4.84	0.74	4.75	1.15	5.11	1.47
GC-rich 0.5M	6.12	1.37	5.94	1.37	5.90	1.54
GC-rich 1.5M	6.13	1.18	5.60	1.35	5.88	1.56
