# Supplementary material for: Molecular basis of Arginine and Lysine DNA sequence-dependent thermo-stability modulation
Source: PLoS Comput Biol. 2022 Jan 10;18(1):e1009749. doi: 10.1371/journal.pcbi.1009749 (PMC8782489; doi:10.1371/journal.pcbi.1009749)
Supplement: S4 Table — (PDF) [file pcbi.1009749.s004.pdf]

S4 Table. Averaged overall stiffness values for the entire duplex (in kJ/(mol).

	AT-rich			GC-rich		
	Na	ZR	ZK	Na	ZR	ZK
25mM	16.09	17.50	16.49	15.69	16.10	16.18
500mM	14.96	19.30	18.98	15.93	19.59	17.54
1500mM	16.58	20.45	19.63	16.02	19.10	17.55
