# Supplementary material for: Molecular basis of Arginine and Lysine DNA sequence-dependent thermo-stability modulation
Source: PLoS Comput Biol. 2022 Jan 10;18(1):e1009749. doi: 10.1371/journal.pcbi.1009749 (PMC8782489; doi:10.1371/journal.pcbi.1009749)
Supplement: S5 Table — (PDF) [file pcbi.1009749.s005.pdf]

S5 Table. Normalized overlap of the covariance matrices computed using the 10 first eigenvectors.

AT-rich	Na 25mM	Lys 25mM	Arg 25mM	Na 500mM	Lys 500mM	Arg 500mM	Na 1500mM	Lys 1500mM	Arg 1500mM
Na 25mM		0.969	0.972	0.972	0.959	0.942	0.968	0.955	0.952
Lys 25mM			0.967	0.962	0.958	0.934	0.965	0.956	0.953
Arg 25mM				0.966	0.965	0.944	0.960	0.957	0.959
Na 500mM					0.954	0.935	0.962	0.952	0.945
Lys 500mM						0.955	0.953	0.965	0.965
Arg 500mM							0.935	0.949	0.960
Na 1500mM								0.959	0.949
Lys 1500mM									0.960
Arg 1500mM									

GC-rich	Na 25mM	Lys 25mM	Arg 25mM	Na 500mM	Lys 500mM	Arg 500mM	Na 1500mM	Lys 1500mM	Arg 1500mM
Na 25mM		0.981	0.978	0.981	0.966	0.959	0.980	0.965	0.963
Lys 25mM			0.976	0.979	0.967	0.956	0.978	0.967	0.963
Arg 25mM				0.978	0.969	0.963	0.981	0.967	0.957
Na 500mM					0.964	0.962	0.980	0.966	0.960
Lys 500mM						0.947	0.971	0.970	0.955
Arg 500mM							0.959	0.951	0.944
Na 1500mM								0.969	0.956
Lys 1500mM									0.951
Arg 1500mM									
