# Supplementary figures and images for: Molecular basis of Arginine and Lysine DNA sequence-dependent thermo-stability modulation
Source: PLoS Comput Biol. 2022 Jan 10;18(1):e1009749. doi: 10.1371/journal.pcbi.1009749 (PMC8782489; doi:10.1371/journal.pcbi.1009749)

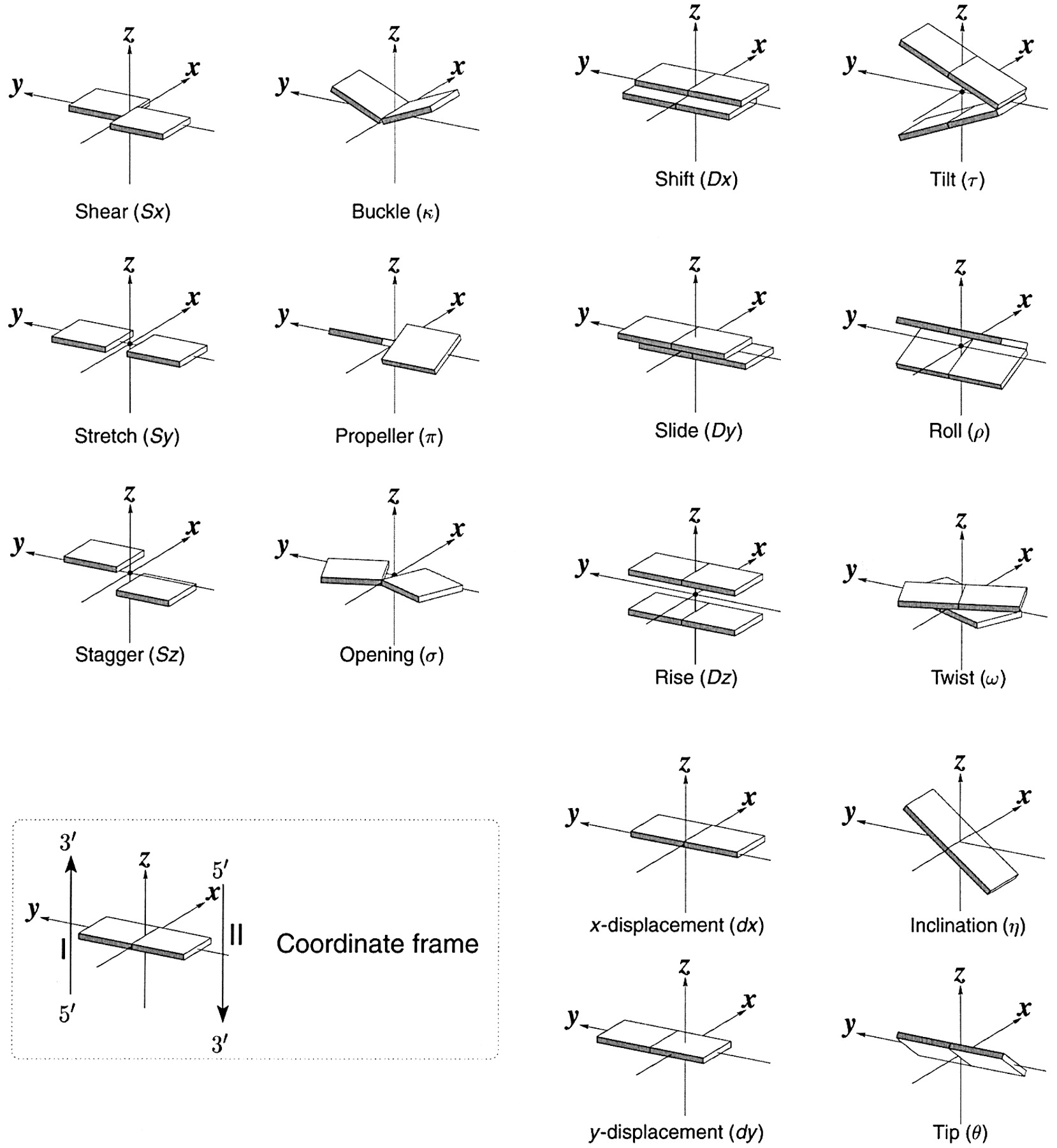

Supplement: S1 Fig — Adapted from Lu and Olson 2003 [2]. (TIF) [file pcbi.1009749.s006.tif]

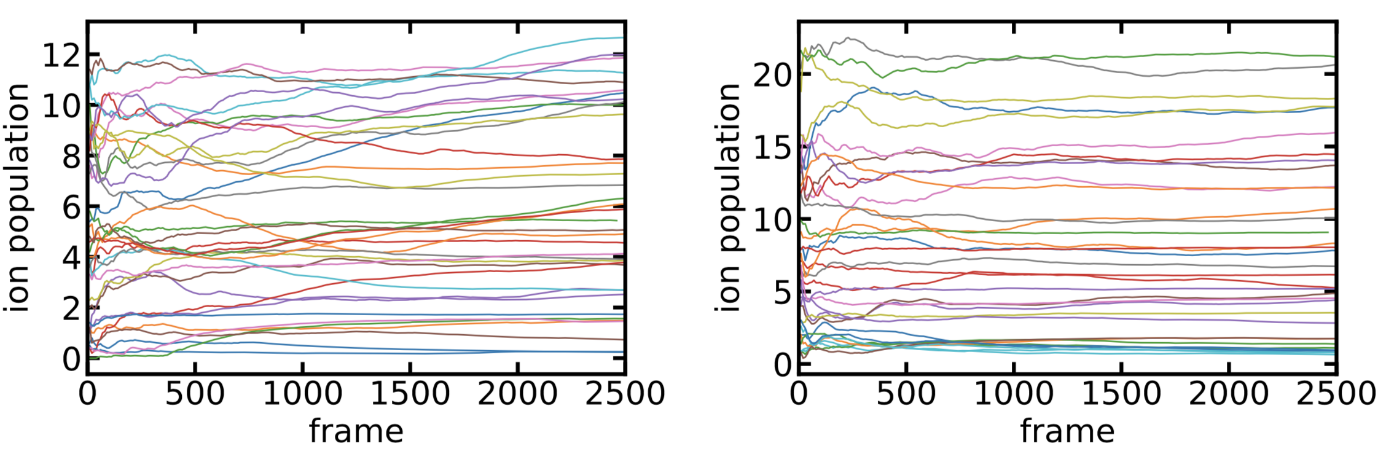

Supplement: S2 Fig — Left figure. Inner minor-groove Right figure. Inner major-groove. Note that here 2,500 frames are equivalent to 5 microseconds. (TIF) [file pcbi.1009749.s007.tif]

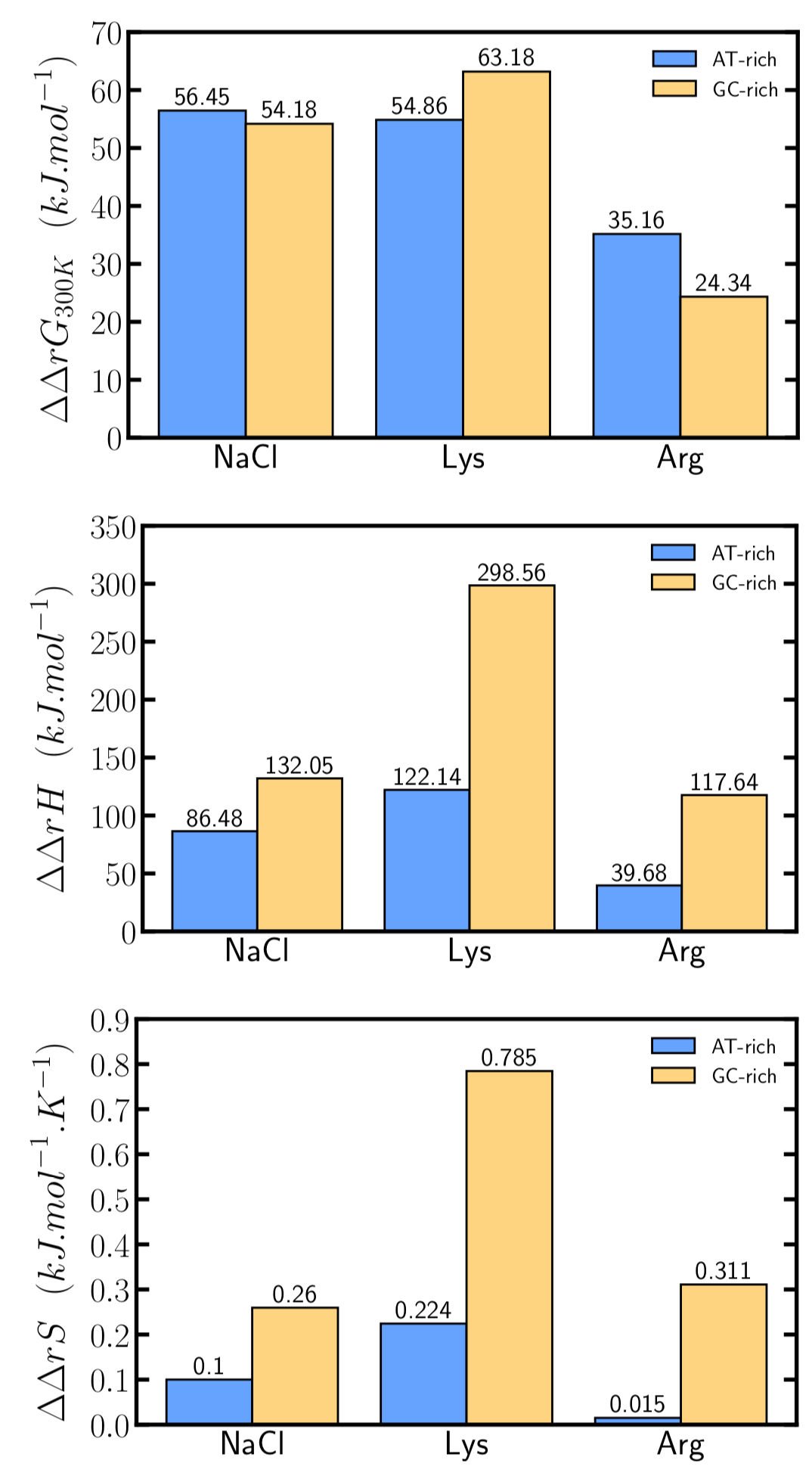

Supplement: S4 Fig — TOP: ΔΔrGcondition−NaP,300°K for each cationic condition. MIDDLE: ΔΔrHcondition−NaP for each cationic condition. BOTTOM: ΔΔrScondition−NaP for each cationic condition. All the values are related to the direct DNA duplex melting reaction. (TIF) [file pcbi.1009749.s009.tif]

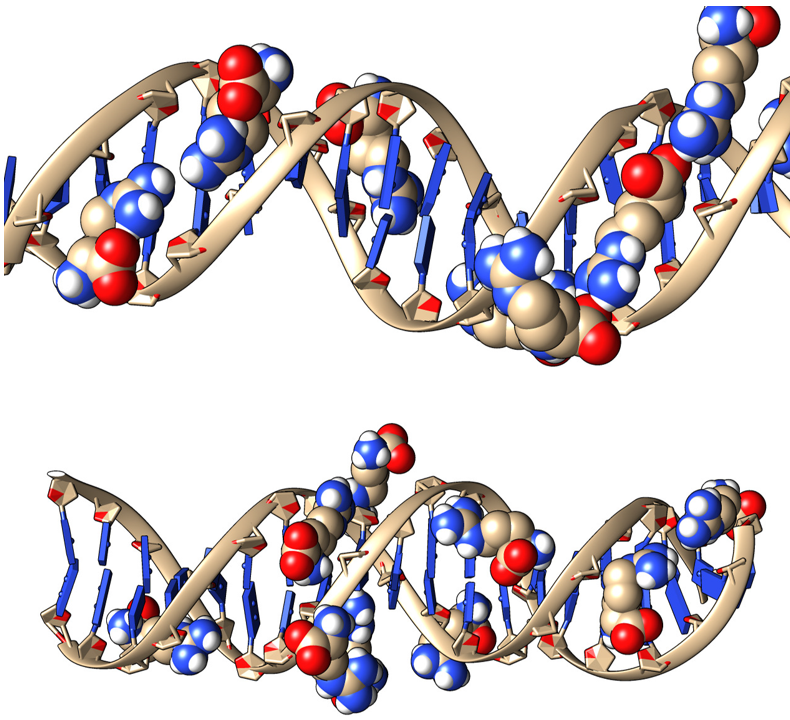

Supplement: S5 Fig — 25mM MD simulations were used to extract this Figure. (TIF) [file pcbi.1009749.s010.tif]

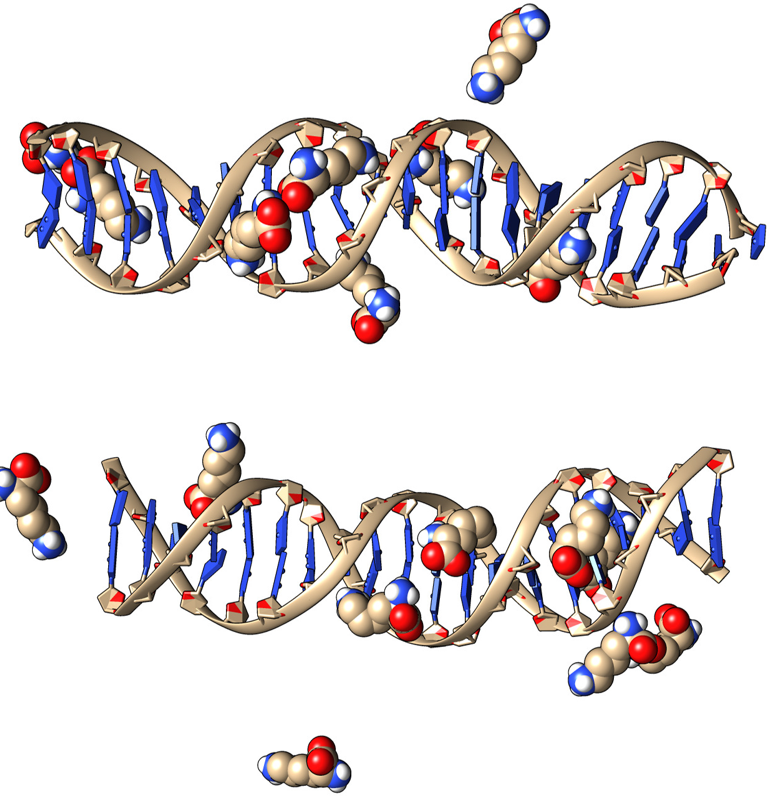

Supplement: S6 Fig — 25mM MD simulations were used to extract this Figure. (TIF) [file pcbi.1009749.s011.tif]

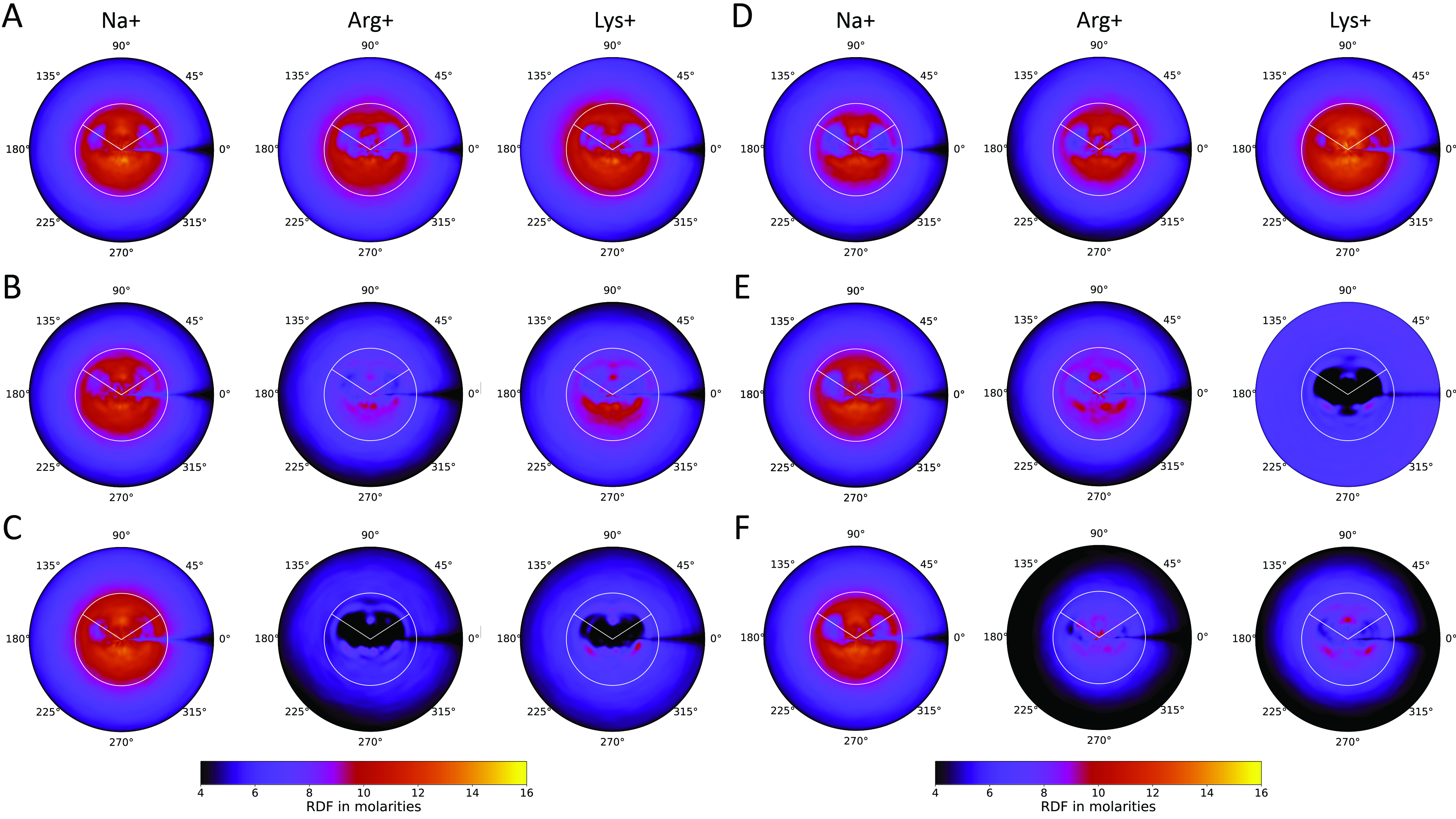

Supplement: S7 Fig — A) Averaged radial-angular dependence of OW atom from AT-rich duplexes at 25 mM concentration. The centre of each circle represent the average axis of the DNA-duplex. The minor-groove extends from 33° to 147°, while the major-groove extends from 147° to 33° (in the trigonometric direction). The white circle delimits the inner space of both minor and major grooves (localized 10.25 Å from the DNA average axis). B) Same as (A) for simulations done at 500 mM. C) Same than (B) at 1.5 M concentration. D), E), F), same as (A), (B), and (C) for GC-rich DNA duplexes respectively. (TIF) [file pcbi.1009749.s012.tif]

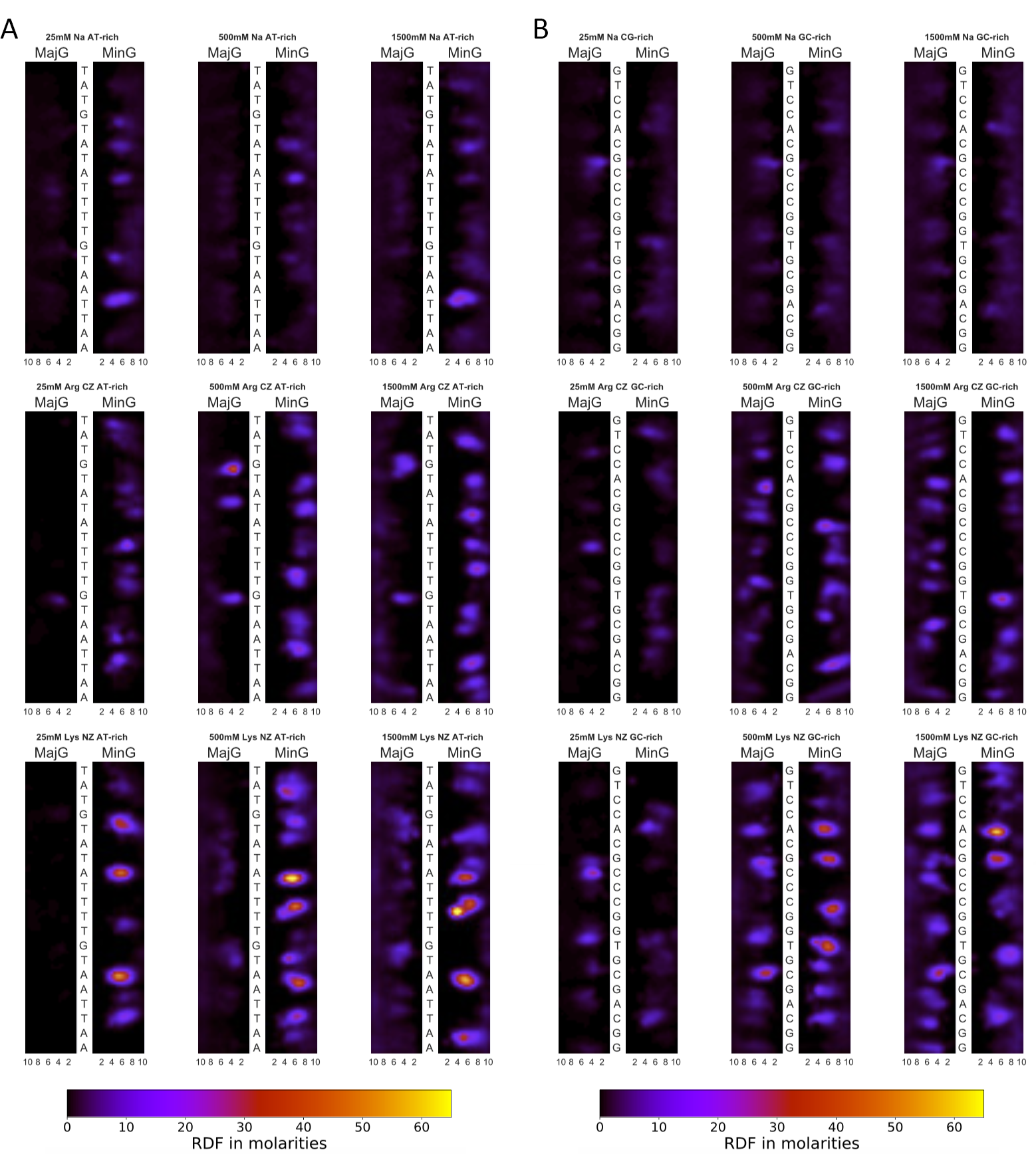

Supplement: S8 Fig — Radial distribution function of the cations around AT- (A) and GC-rich (B) DNA duplexes considering the Na, CZ or NZ atoms. The x-axis represents the distance from DNA average axis in Å ranging from 0 to 10.25. The sequence in place of the DNA average axis is the arbitrary Watson sequence as written in Material and Methods. MajG: major-groove. MinG: minor-groove. (TIF) [file pcbi.1009749.s013.tif]

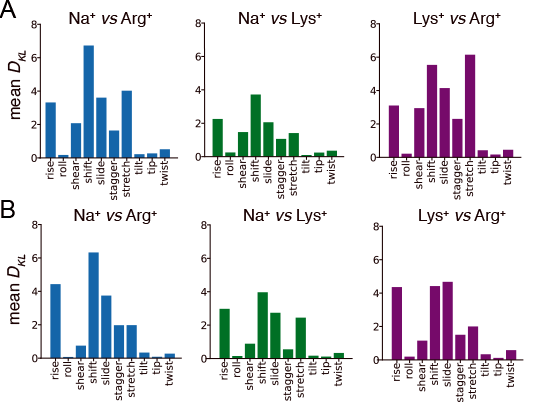

Supplement: S9 Fig — KL-divergence between helical parameters for each cationic conditions at 1500mM ion concentration for AT- (A) and GC-rich (B) DNA duplexes. Note that only the helical parameters that showed some changes are reported. (TIF) [file pcbi.1009749.s014.tif]

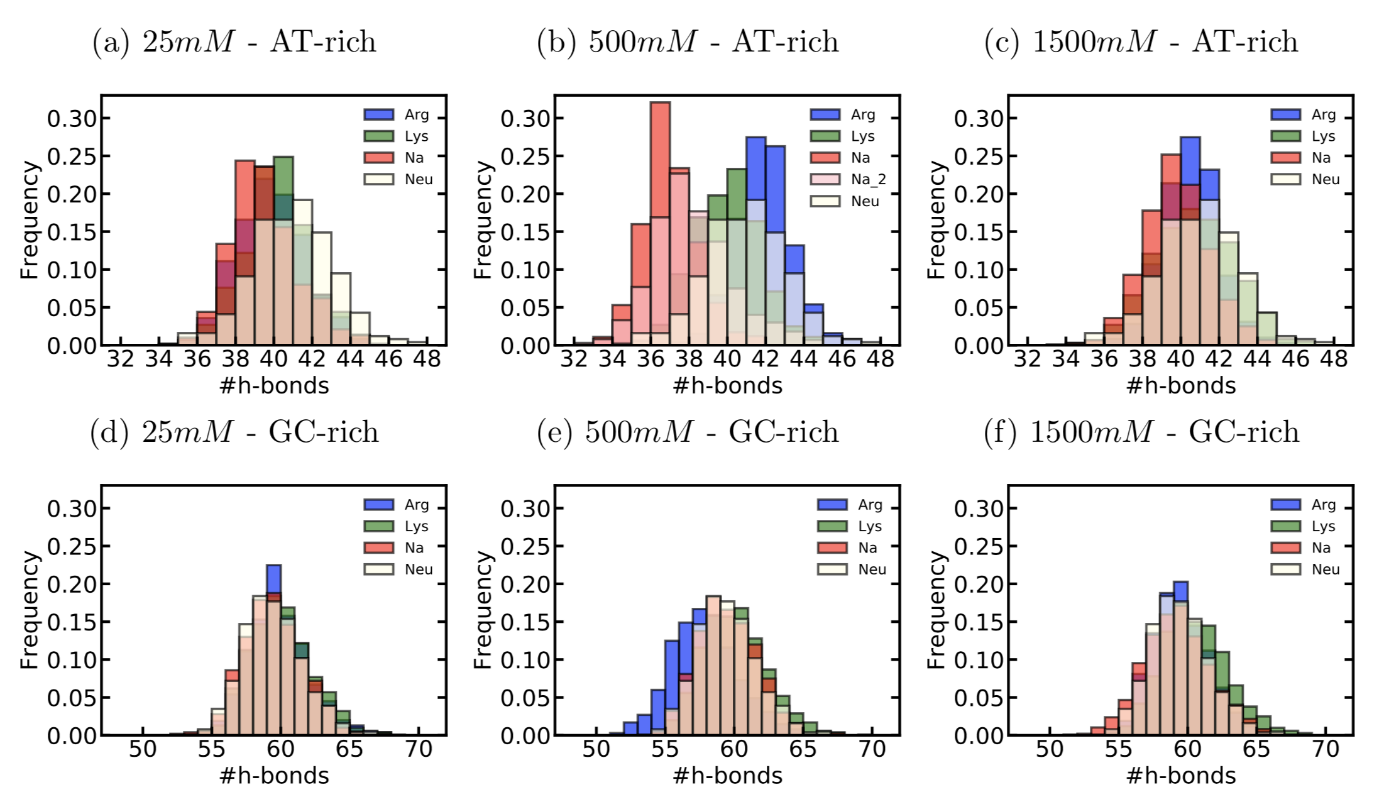

Supplement: S10 Fig — Only the final 200ns of the MD trajectories were considered. (TIF) [file pcbi.1009749.s015.tif]

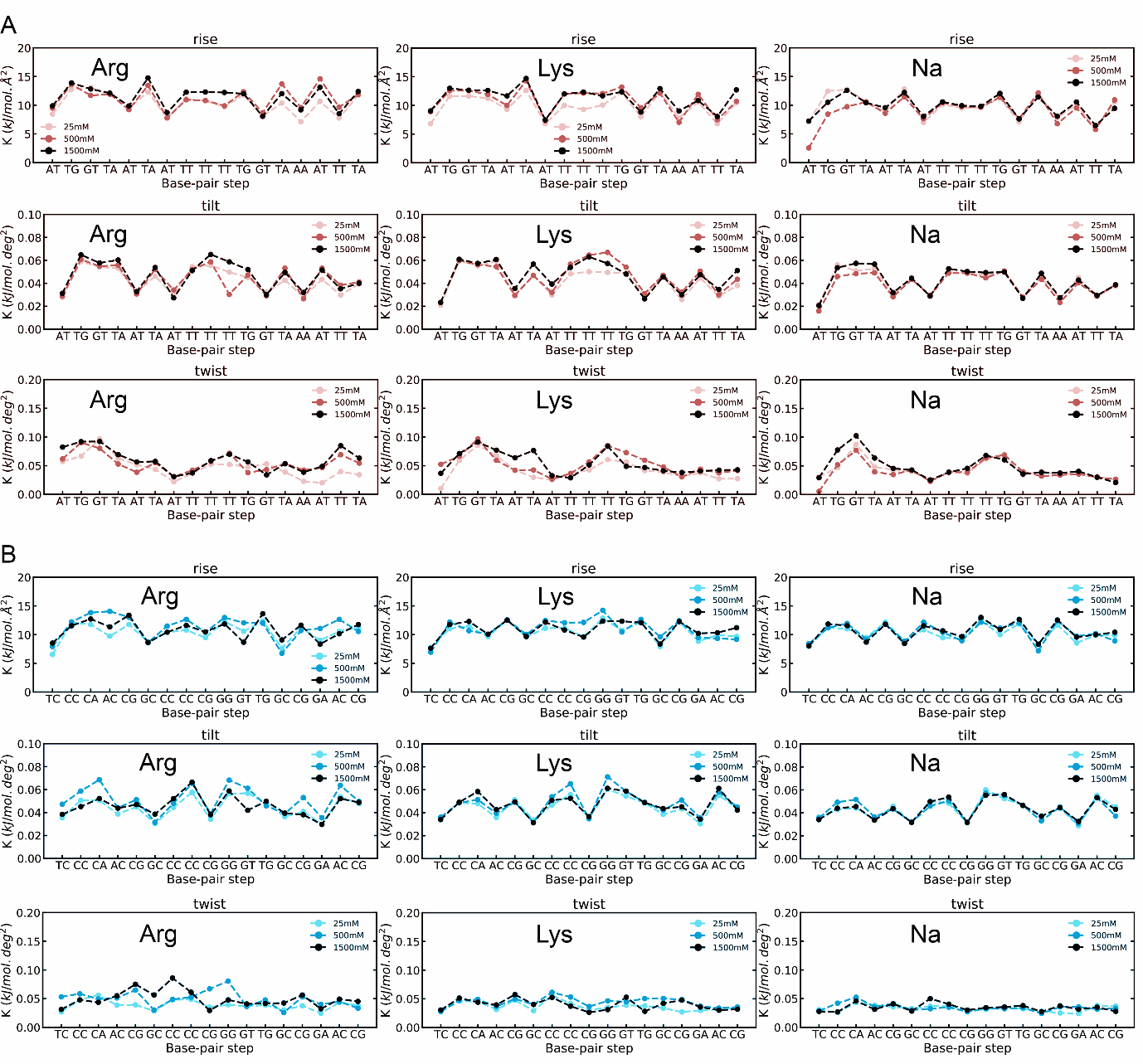

Supplement: S11 Fig — A) Pure (diagonal values) of Rise-Rise, Tilt-Tilt and Twist-Twist sequence-dependent force constants for AT-rich sequences (red-ish series). B) Same as (A) for GC-rich sequences (blue-ish series). (TIF) [file pcbi.1009749.s016.tif]

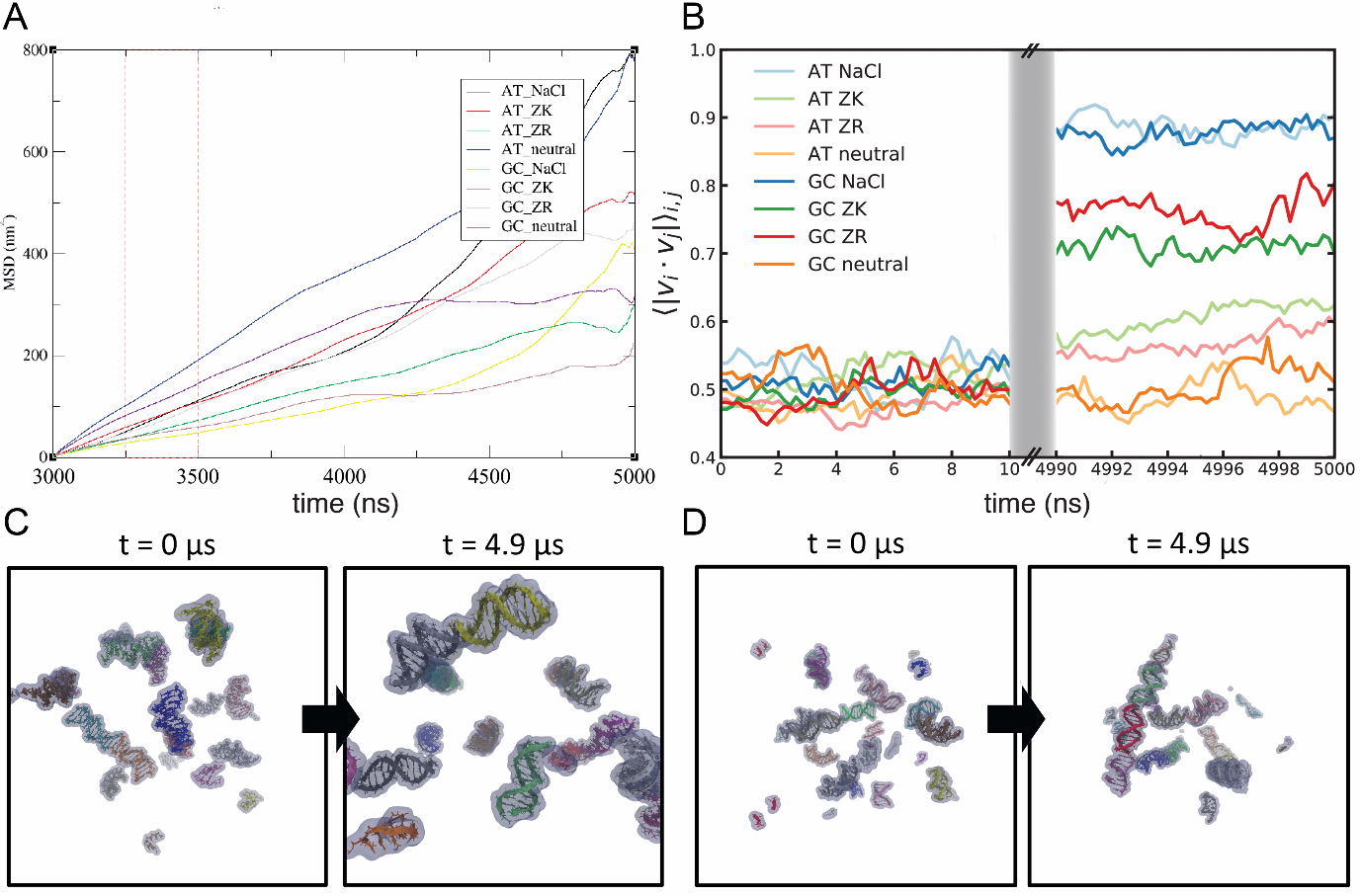

Supplement: S12 Fig — A) Mean-Square-Displacement (MSD) along the simulated time. Note that the Diffusion Coefficient was computed from 3.25 μs to 3.5 μs (linear regime between the vertical dashed lines). B) Pair-wise cross-correlation coefficient between vectors that represents the helical axe of each DNA duplex along the total simulated time. First and last 10 ns are shown. C) Initial and one representative “final” structure obtained from the corresponding MD trajectories of AT-rich systems under electroneutrality conditions. Each duplex is depicted in cartoon representation using different colors. D) Same as (C) for GC-rich systems. (TIF) [file pcbi.1009749.s017.tif]
